# Density Functional Theory Investigation of Temperature-Dependent Properties of Cu-Nitrogen-Doped Graphene as a Cathode Material in Fuel Cell Applications

**DOI:** 10.3390/molecules28237873

**Published:** 2023-11-30

**Authors:** Yashas Balasooriya, Pubudu Samarasekara, Chee Ming Lim, Yuan-Fong Chou Chau, Muhammad Raziq Rahimi Kooh, Roshan Thotagamuge

**Affiliations:** 1 Postgraduate Institute of Science, University of Peradeniya, Peradeniya 20400, Sri Lanka; yashasbalasooriya@gmail.com; 2 Department of Physics, Faculty of Science, University of Peradeniya, Peradeniya 20400, Sri Lanka; pubudus@sci.pdn.ac.lk; 3 Centre for Advanced Material and Energy Sciences, Universiti Brunei Darussalam, Jalan Tungku Link, Gadong BE 1410, Brunei; cheeming.lim@ubd.edu.bn (C.M.L.); chou.fong@ubd.edu.bn (Y.-F.C.C.); 4 Department of Nano Science Technology, Faculty of Technology, Wayamba University of Sri Lanka, Kuliyapitiya 60200, Sri Lanka

**Keywords:** DFT, fuel cell, oxygen reduction reaction, temperature effect on fuel cell, H_2_O_2_ generation, copper–nitrogen-doped graphene

## Abstract

In this study, density functional theory (DFT) was used to investigate the influence of temperature on the performance of a novel Cu-nitrogen-doped graphene Cu_2_-N_8_/Gr nanocomposite as a catalyst for the oxygen reduction reaction (ORR) in fuel cell applications. Our DFT calculations, conducted using Gaussian 09w with the 3–21G/B3LYP basis set, focus on the Cu-nitrogen-doped graphene nanocomposite cathode catalyst, exploring its behavior at three distinct temperatures: 298.15 K, 353.15 K, and 393.15 K, under acidic conditions. Our analysis of formation energies indicates that the structural stability of the catalyst remains unaffected as the temperature varies within the potential range of 0–7.21 V. Notably, the stability of the ORR steps experiences a marginal decrease with increasing temperature, with the exception of the intermediate OH + H_2_O (*OH + H + *OH). Interestingly, the optimization reveals the absence of single OH and H_2_O intermediates during the reactions. Furthermore, the OH + H_2_O step is optimized to form the OH + H + OH intermediate, featuring the sharing of a hydrogen atom between dual OH intermediates. Free energy calculations elucidate that the catalyst supports spontaneous ORR at all temperatures. The highest recorded maximum cell potential, 0.69 V, is observed at 393.15 K, while the lowest, 0.61 V, is recorded at 353.15 K. In particular, the Cu_2_-N_8_/Gr catalyst structure demonstrates a reduced favorability for the H_2_O_2_ generation at all temperatures, resulting in the formation of dual OH intermediates rather than H_2_O_2_. In conclusion, at 393.15 K, Cu_2_-N_8_/Gr exhibits enhanced catalyst performance compared to 353.15 K and 298.15 K, making it a promising candidate for ORR catalysis in fuel cell applications.

## 1. Introduction

Fuel cells operating at low temperatures have attracted considerable attention due to their enhanced durability, reliability, and efficiency with low weight, making them more portable compared to their counterparts. These cells typically operate at temperatures below 473.15 K, often around 373.15 K, and commonly rely on carbon-supported catalysts to enhance the oxygen reduction reaction (ORR). However, these carbon supports are susceptible to rapid corrosion at higher temperatures, resulting in catalyst agglomeration, material durability, and subsequent reductions in efficiency [1].

Walch et al. revealed the effectiveness of the DFT method in studying ORR performance and catalyst properties [2]. Commercially available low-temperature fuel cells predominantly employ platinum group metals (PGMs), such as platinum (Pt), palladium (Pd), and iridium (Ir), as catalysts. These costly materials effectively facilitate hydrogen ionization at the anode [3,4] and provide an efficient ORR surface at the cathode [5]. However, as the carbon support undergoes oxidation, the catalyst layered upon it tends to agglomerate, resulting in reduced fuel cell efficiency over short operational cycles. Catalyst materials made of transition-metal-based perovskite oxides have also been used as effective catalysts compared to PGM. However, the process of creating those materials is relatively costly due to the need for La, Sr-like alkaline-earth/rare-earth materials, and transition metals [6]. To address this issue and lower the costs, non-platinum group metals (non-PGM) have been explored as potential catalysts. These alternatives, typically composed of nitrogen atoms doped onto graphite layers [7,8,9] or transition metal layers [10], offer advantages such as ease of fabrication, high electron conductivity, and cost-effectiveness [11,12,13].

Low-temperature fuel cells, such as proton exchange membrane (PEM) fuel cells, have shown improved efficiency with increasing temperature [14]. A temperature range of 353.15–403.15 K has been identified as particularly promising for low-temperature fuel cells [15]. Some studies have reported significant performance enhancements in PEM fuel cells at a temperature of 393.15 K [15,16]. Raising the temperature has the benefits of increased voltage and efficiency, but it also introduces the drawback of reduced durability [14,16,17].

The output voltage, as per the Nernst equation, is directly proportional to the temperature, with a higher temperature resulting in enhanced charge particle kinetics and an increased fuel cell voltage [18]. However, the lowest performance of the fuel cell is observed at around 343.15 K [19]. As Guan et al. explained, the reason for the lowest performance at 343.15 K and the highest performance at 393.15 K may be a phase change that occurs with increasing temperature. Furthermore, as temperature increases, the oxygen evolution reaction (OER) takes place, leading to the reconstruction of the catalyst surface [20]. To achieve a voltage increase, the enhancement in voltage must exceed the voltage loss due to the negative thermodynamic correlation between the open-circuit voltage and temperature [18,21].

The rate of catalyst decay is influenced by several factors, including temperature, ORR, hydrogen oxidation reaction (HOR), high potential, and the pH of the medium [14]. The formation of carbon monoxide (CO) at the cathode is due to the reaction between water and the carbon support of the catalyst that can cover the catalyst, and this significantly reduces the electrochemical reaction [22]. This process takes place mainly at temperatures below 373.15 K [23]. Above 373.15 K, the water generated from the reaction turns into vapor and is rapidly removed. This dehydration process causes a reduction in CO generation from the catalyst [24].

Numerous non-PGM catalysts, such as Fe/N/Gr, Co/N/Gr, and Mn/N/Gr, have been extensively studied, and some have even reached commercialization [25,26]. For instance, Mn/N/Gr catalysts have been demonstrated to be unstable for potential above 0.53 V [27], while Co/N/Gr-based catalysts tend to produce hydrogen peroxide (H_2_O_2_) during ORR, which reduces the current density and can damage the fuel cell membrane [28]. In contrast, Fe-based non-PGM catalysts have been shown to be promising, especially with dual metal structures such as Fe_2_-N_6_/Gr and Fe_2_-N_8_/Gr [29,30]. Increasing the Fe content to 1.0 wt % can significantly reduce H_2_O_2_ generation [31]. However, a drawback of Fe-based catalysts is the formation of Fe(OH)_2_ during ORR, which can lead to the removal of Fe atoms from the catalyst layer [32]. Furthermore, Fe oxidation can contribute to the catalyst layer degradation [33].

Previous research on copper-based non-PGM single metal catalysts has shown them to have good spontaneous ORR [34,35]. In our previous work, we performed a DFT investigation on Cu-based novel duel metal non-PGM catalyst structures (Cu_2_-N_6_/Gr and Cu_2_-N_8_/Gr) and a single metal structure (Cu-N_4_/Gr) [36], and our study showed that all three structures exhibit higher stability than the previously studied Cu-N_2_/Gr structure. The study also showed that the possibility of H_2_O_2_ formation is much less with dual metal structures compared to single metal structures. It is noted that the study was carried out at an initial temperature of 298.15 K and pressure of 1 atm without applying van der Waals force correction. In conclusion, the Cu_2_-N_8_/Gr structure showed promising results, confirming its potential for high cell potential with spontaneous ORR and the absence of H_2_O_2_ formation. Additionally, Cu-N_4_/Gr was also shown to have a high cell potential with spontaneous ORR but has a higher probability of forming H_2_O_2_.

In this study, DFT calculations were performed to determine the effect of temperature on the Cu_2_-N_8_/Gr catalyst in an acidic medium. To avoid the limitations of previous studies, a dispersion correction was applied to the DFT calculation. Salam et al., Amrit et al., and Ferraris et al. have reported that the performance of low-temperature fuel cells changes dramatically at temperatures around 298.15, 353.15, and 393.15 K due to changes in the particle kinetics with temperature using experimental methods, as previously stated [14,15,19]. Therefore, for this study, 298.15, 353.15, and 393.15 K temperature values were selected to investigate the impact of temperature on the fuel cell performance of Cu_2_-N_8_/Gr as a cathode catalyst in an acidic medium. At these three temperature values, the structural stability, binding ability of ORR steps, the break-free ability of H_2_O, evidence of H_2_O_2_ generation, and maximum cell potential were predicted as assessments of objectives.

## 2. Results and Discussion

### 2.1. Stability and Reactivity

Stability and reactivity were predicted using formation energy data [28,35], and the HOMO–LUMO energy calculation [37,38,39,40] is elaborated in Equation (6) and Equations (15)–(18), respectively. Under open circuit conditions, the Cu_2_-N_8_/Gr structure provided formation energy values of −28.856, −28.865, and −28.864 eV for the three different temperatures (298.15, 353.15, and 393.15 K) at zero potential (*U* = 0), as presented in Table 1. According to Equation (6), an increase in the potential value leads to an enhancement of the formation energy, resulting in a decrease in the structure’s stability. Minor changes in formation energy, at the same potential, generated due to thermal correction were small in comparison to the optimized energy. Negative values in the formation energies indicate that the energy gained favors structural stability. Notably, the formation energy of the structure at all the selected temperatures remained negative for external potentials ranging between 0 and 7.21 V. According to free energy data, the highest maximum cell potential of 0.69 V is observed under an external potential of 7.21 V, which favors this situation. Table 1 illustrates the results showing that the catalyst structure remains stable at all three temperatures as long as the external potential values do not exceed 7.21 V.

**Figure 1:**
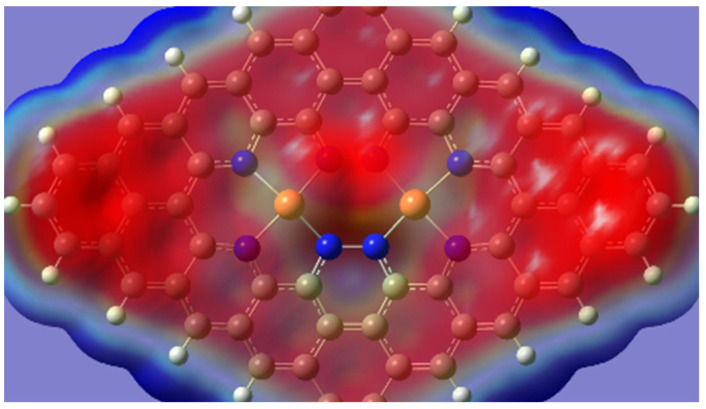
Electrostatic potential diagram of the defect structure at all temperatures (red and blue areas represent the negative and positive charges, respectively, and the increasing density of the color represents the increasing charge density).

In the case of the dual metal structure, Cu_2_-N_8_/Gr involves multiple copper atoms that coordinate with eight nitrogen atoms. This configuration results in a significantly more extensive bonding network. It enhances coordination stability when compared to single metal structures, such as Cu-N_4_/Gr and Cu-N_2_/Gr, in which only one metal atom coordinates with a smaller number of nitrogen atoms. This increased stability is compatible with findings reported by Yang et al. [41]. Additionally, the dual metal structure is likely to facilitate a more efficient distribution and delocalization of electrons due to the presence of multiple Cu and N atoms. The electrostatic potential (ESP) diagram (Figure 1), which supports the electron distribution theory, for all three temperatures, shows no significant differences in electronic structure. The red area represents the distribution of negative charge (electrons), and the blue and white areas represent positive charge and depleted charge density, respectively.

HOMO–LUMO energy calculations, as explained in Equations (15)–(18), were carried out to investigate the stability and reactivity of the Cu_2_-N_8_/Gr and *O_2_, *H_2_O, and OH + H_2_O intermediates [37,38,39,40]. Specifically, the energy gap (*E*_g_), chemical hardness (*η*), chemical potential (*μ*), and electrophilicity index (*ω*) were calculated for these structures. Here, the *O_2_ and 2*H_2_O intermediates were considered for the initial and final ORR steps, respectively. These intermediates are important when compared to other ORR steps due to the breaking of the O_2_ bond with the catalyst that initiates the ORR, and 2H_2_O is released from the catalyst to conclude the ORR. The OH + H_2_O intermediate was also considered due to its abnormality. Within all the temperature ranges considered, the HOMO–LUMO calculations remained constant, except for the OH + H_2_O intermediate, indicating that the catalyst structure did not change for the given temperatures.

**Figure 2:**
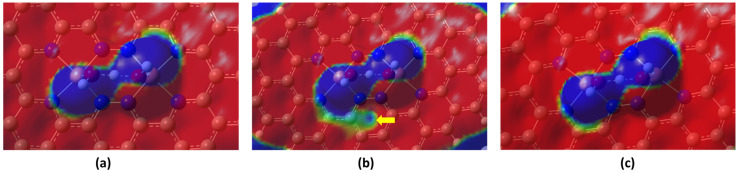
Electrostatic potential diagram of the OH + H_2_O intermediate at the temperatures of (**a**) 298.15 K (**b**) 353.15 K, and (**c**) 393.15 K (red and blue areas represent the negative and positive charges, respectively, and increasing density of color represents increasing charge density).

**Figure 3:**
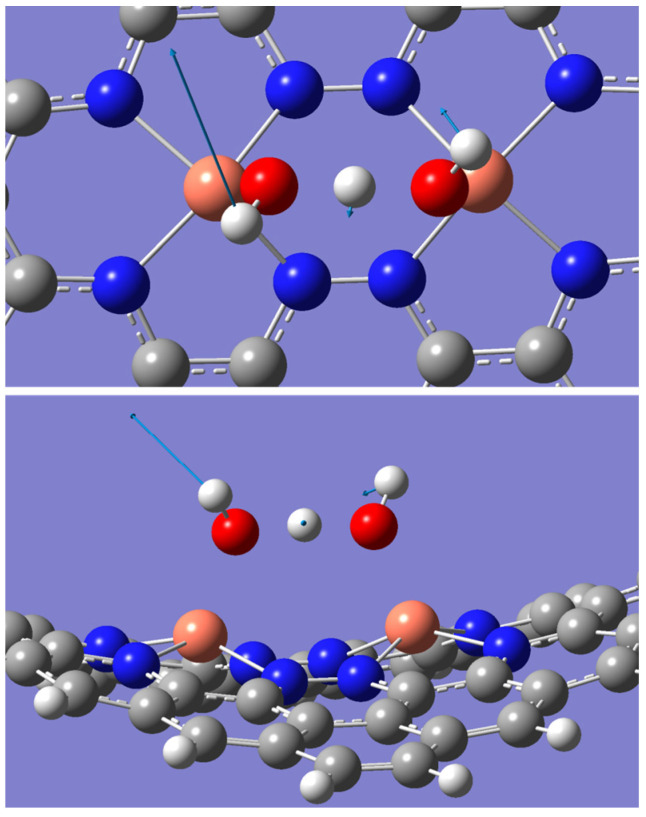
Top and side displacements of the *OH + H + *OH intermediate around the 289.32 cm^−1^ frequency according to vibrational data at 353.15 K (white, grey, blue, orange, and red spheres represent hydrogen, carbon, nitrogen, copper, and oxygen atoms, respectively).

Chemical hardness is a measure that reflects the stability and reactivity of a structure, where stable structures tend to be less reactive, and highly reactive structures are typically less stable [9,38,42]. Table 2 shows that the OH + H_2_O intermediate’s stability increases in the following temperature order: 353.15 K < 298.15 K < 393.15 K. This indicates that at 393.15 K, the intermediate becomes more stable, while at 353.15 K it becomes highly reactive compared to the other temperatures. The ESP diagram in Figure 2 reveals that as the temperature increases from 298.15 to 353.15 K, the electron charge density decreases around the Cu-N structure. The pointed green area in Figure 2b indicates the potential depletion of the electron density. This causes the lower stability of the OH + H_2_O intermediate at 353.15 K. However, when the temperature reaches 393.15 K, this depletion area is significantly reduced. Furthermore, at 393.15 K, the electron distribution becomes more symmetric (Figure 2c) around the Cu-N structure, as indicated by the similarly sized green area on the opposite side of the blue area. The high stability of the OH + H_2_O intermediate at 393.15 K is attributed to this symmetric electron charge distribution. According to Watanabe et al., vibrational motion can significantly change the electron density [43], and as the temperature increases, the vibration frequency and direction of the atoms change rapidly. This is attributed to variations in the ESP at different temperatures. To confirm the vibration effects, the vibration modes at different frequencies were examined at a temperature of 353.15 K. A frequency around 289.32 cm^−1^ indicates the displacement of H atoms, as shown in Figure 3. This mode also has a significantly high infrared value of 25.30 compared to other frequencies. Other vibrational modes were considered but then neglected because their vibration direction did not align with the charge distribution in the ESP diagram. Here, the H atoms represent the most positive charge in the ESP diagram. Therefore, the long displacement vector of the H atom (left H atom) compared to the abnormal electron depletion area is highlighted in Figure 2b. The displacement vector of the hydrogen atom located on the right-hand side in Figure 3 exhibits a smaller magnitude in comparison to the hydrogen atom situated on the left-hand side, an observation consistent with the electrostatic potential (ESP) depicted in Figure 2b. The middle H atom, with the smallest displacement vector, creates a bottleneck in the dumbbell-shaped electron-depletion area in the ESP diagram.

The chemical reactivity of the structure increases as the chemical potential decreases. Chattaraj et al. [38] established a direct connection between Mulliken charge negativity and chemical potential. For all temperatures, the chemical potentials of the O_2_ and 2H_2_O intermediates, and the Cu_2_-N_8_/Gr structure remain unchanged. The chemical potentials of O_2_ and 2H_2_O, which are −3.615 and −3.322 eV, respectively, indicate that O_2_ has higher reactivity than H_2_O. Therefore, the optimized 2H_2_O intermediate is more stable, suggesting that it can easily detach from the catalyst structure due to its weak bond strength and long bond length. The chemical potential of the OH + H_2_O intermediate decreases in the following order for different temperatures: 298.15 K > 393.15 K > 353.15 K. This order aligns with the high reactivity of the OH + H_2_O intermediate observed in the chemical hardness data.

The electrophilicity indices of O_2_ and 2H_2_O at all temperatures are 8.854 and 8.048 eV, respectively. Since the electrophilicity index represents the ability to attract electrons from the surroundings to form a stable structure, the O_2_ intermediate is more reactive than 2H_2_O, as explained in the chemical potential data. For the OH + H_2_O intermediate, the lowest and highest electrophilicity indices are observed at temperatures of 393.15 and 353.15 K, respectively, which is in agreement with the chemical hardness data. The order of reactivity for the OH + H_2_O intermediate is as follows for different temperatures: 353.15 K > 298.15 K > 393.15 K, which confirms the chemical potential data, indicating its highest reactivity at 353.15 K.

**Figure 4:**
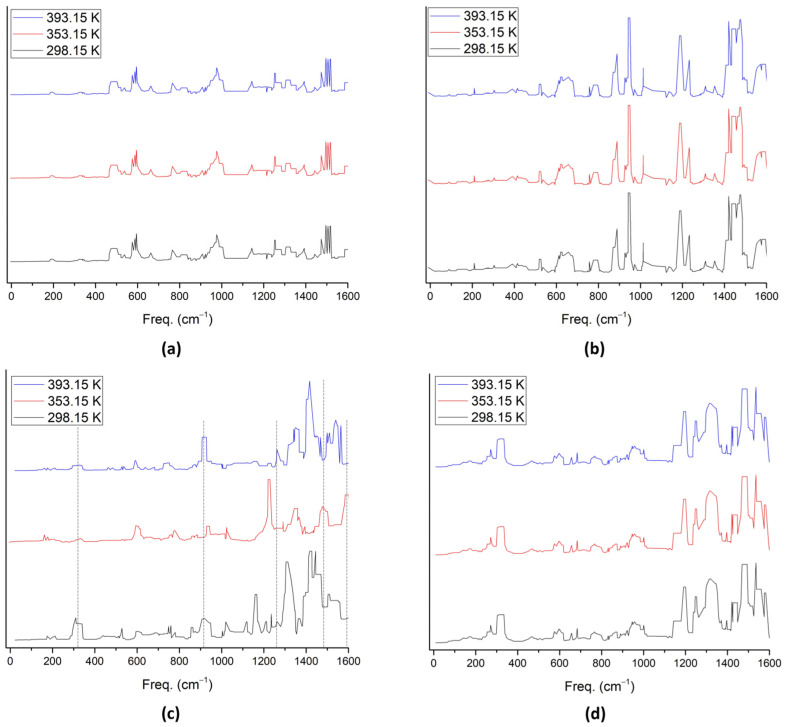
IR spectra of different temperatures for (**a**) the catalyst (*), (**b**) *O_2_, (**c**) OH + H_2_O, and (**d**) H_2_O + H_2_O.

The infrared (IR) spectra, illustrated in Figure 4a–d, were examined for the catalyst (*), *O_2_, *OH + H_2_O, and *H_2_O + H_2_O. Notably, the peaks remained unchanged with variations in temperature, except for *OH + H_2_O, as depicted in Figure 4c. This consistent behavior in the IR spectrum substantiates stability across temperature variations and aligns with the results obtained from other calculations, such as HOMO–LUMO energy calculations assessing stability and reactivity.

Moreover, Figure 4c highlights that at temperatures of 298.15 K and 393.15 K, similar peaks are observed. However, at 353.15 K, shifts or variations in peaks are evident, indicating distinct behaviors of the *OH + H_2_O intermediate at this temperature. Simulation results suggest vibrational activity around the frequency of 289.32 cm^−1^ for the OH + H_2_O intermediate, while higher peaks signify vibrations of C and H atoms at the catalyst layer’s edge.

Furthermore, at 353.15 K, certain peaks of the OH + H_2_O intermediate exhibit a shift towards lower wavenumbers within the range of 1200–1600 cm^−1^. This observation implies thermal denaturation, as explained by Panick et al. [44], or a phase change associated with temperature, as elucidated by Guan et al. [20]. These findings contribute valuable insights into the temperature-dependent dynamics of the studied OH + H_2_O intermediate.

### 2.2. ORR Steps and Binding Energy

**Figure 5:**
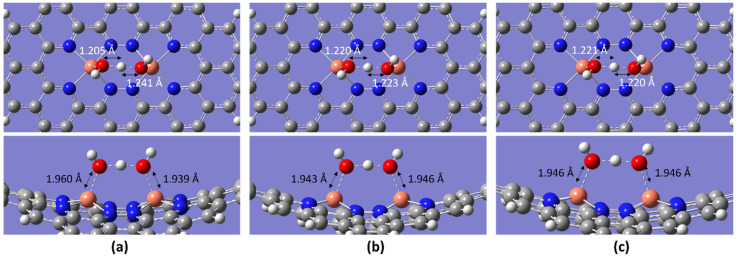
Optimized intermediate of OH + H_2_O (*OH + H + *OH) at the temperatures of (**a**) 298.15 K (**b**) 353.15 K, and (**c**) 393.15 K (white, grey, blue, orange, and red spheres represent hydrogen, carbon, nitrogen, copper, and oxygen atoms, respectively).

In this study, the ORR steps did not indicate deviations at different temperatures. However, when the intermediate 2OH is bonded with an H atom to proceed to the next step, some abnormalities are observed. The expected outcome after *2OH^−^ + H^+^ was *OH with a separated H_2_O molecule. However, after optimization, the *OH + H + *OH intermediate was formed, where both OH molecules shared a single H atom (Figure 5). With the exception of *OH + H + *OH (intermediates after *2OH^−^ + H^+^), the other intermediates did not indicate any deviations in terms of bond lengths, angles, and binding strength with different temperatures (Table 3).

In the first step of ORR, the oxygen molecule exhibits a significant binding strength value compared with that reported by Kattel et al. and Bhatt et al. [28,35], and it is slightly mitigated with increasing temperatures. This trend has been observed for all other intermediates, as recorded in Table 3, except for the OH + H_2_O intermediate (*OH + H + *OH). Initially, the *OH + H + *OH intermediate shows a reduction in its binding strength that mirrors the behavior of the other intermediates. However, at 393.15 K, its binding strength increases compared to the lower temperature of 353.15 K.

Binding energy calculations reveal that the highest binding energy for the OH intermediate was at −6.45 eV, which is observed only at an initial temperature of 298.15 K. At other temperatures, the binding energy significantly decreases to −0.25 eV at 353.15 K and −0.24 eV at 393.15 K. These binding energy values at higher temperatures are not favorable for the ORR, as they represent weaker binding strength for all intermediates. In a typical case, the OH intermediate is generated after the water molecules are rendered and moved away, and subsequently, an H atom attaches itself to the O intermediate, as shown in the ORR reaction Equations (11)–(13). The optimized reactions show that the H atom is attached to the 2OH intermediate, instead of forming a water molecule and OH intermediate. This results in the formation of the OH + H + OH intermediate, as previously explained. Therefore, the weakest bond strength of the OH intermediate is not considered a step in the ORR.

In the final step of the ORR, it is expected that the water molecule will form and easily break away from the catalyst. The calculations confirmed that H_2_O has the lowest binding strength, which favors the described situation. Furthermore, the longest bond length of 2.144 Å between the Cu and O atoms of the catalyst intermediate confirms the weak bond strength. However, according to the optimized ORR steps, the possibility of a single H_2_O intermediate forming at the end of the reaction is not significant. Due to the presence of the OH + H + OH intermediate, the possibility of the formation of a 2H_2_O intermediate is substantial at the end of ORR. The binding energies of the 2H_2_O intermediates are −2.12, −2.11, and −2.10 eV at temperatures of 298.15, 353.15, and 393.15 K, respectively. When compared to the other ORR steps, the 2H_2_O intermediate possesses the weakest bond strength and the longest bond length at all temperatures, favoring the situation.

**Figure 6:**
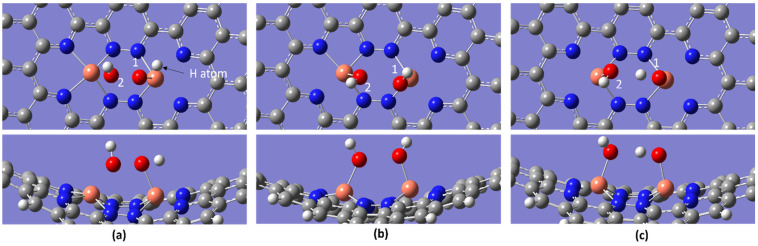
Optimization process when the H atom is introduced to the first O atom of the OOH intermediate at a temperature of 298.15 K. (**a**) Initially, the H atom binds to the first O atom of OOH to form H_2_O_2_, (**b**) in the middle state of optimization, and (**c**) in the final optimization step where dual OH molecules are formed instead of H_2_O_2_ (white, grey, blue, orange, and red spheres represent hydrogen, carbon, nitrogen, copper, and oxygen atoms, respectively).

As the OOH intermediate emerges during the ORR (reaction Equation (2)), there is a significant possibility of H_2_O_2_ generation in the middle of the reaction. To investigate this, the H atom is introduced to the first oxygen atom to determine whether H_2_O_2_ is formed. Figure 6 illustrates this reaction at an initial temperature of 298.15 K. As reported in our previous study [36], under the initial temperature and the other two temperatures, the formation of the 2OH intermediate occurs instead of H_2_O_2_ molecules. This phenomenon can be attributed to the electronegativity of nitrogen (N) being higher than that of copper (Cu). In the Cu_2_-N_8_ structure, where one Cu atom is attached to four N atoms, this arrangement is responsible for the electron cloud depletion around the Cu atoms. Consequently, this can lead to the electrons around the oxygen of the OOH intermediate being highly attracted to Cu atoms. The high binding strengths of the 2OH intermediate, which are −10.97, −8.51, and −8.50 eV at the temperatures of 298.15, 353.15, and 393.15 K, respectively, support this explanation. When a H atom is attached to the first oxygen atom, it can draw electrons away from the first oxygen atom. This effect weakens the O-O bond of the OOH intermediate and leads to the generation of the 2OH intermediate.

**Figure 7:**
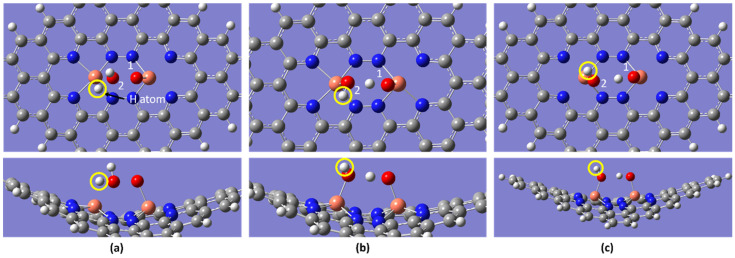
Optimization process when a H atom is introduced to the second O atom of the OOH intermediate at the temperature of 298.15 K: (**a**) initially, the H atom binds with the second O atom of the OOH intermediate to form H_2_O; (**b**) in the next step, the H atom binds to the second O atom, causing the previously bonded H atom to break its bond and migrate to the first O atom; and (**c**) in the final optimization step, a dual OH molecule is formed instead of H_2_O (white, grey, blue, orange, and red spheres represent hydrogen, carbon, nitrogen, copper, and oxygen atoms, respectively).

**Figure 8:**
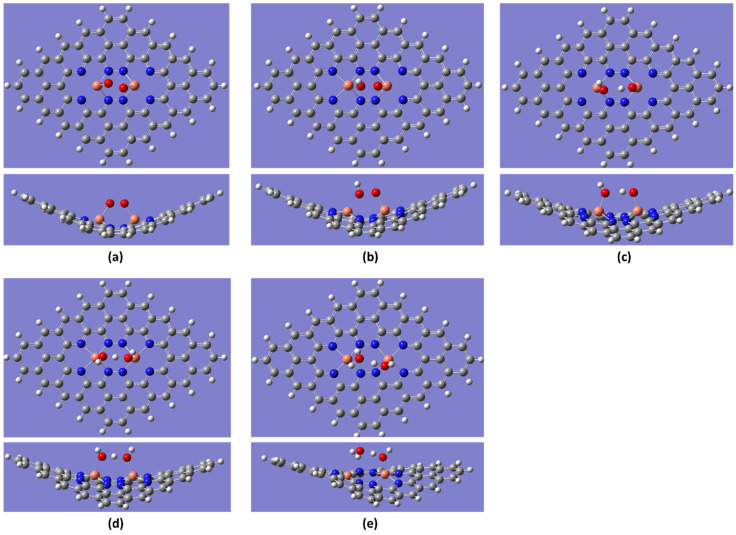
Top and side view of the optimized ORR steps for the Cu_2_-N_8_/Gr structure at 298.15 K. (**a**) O_2_, (**b**) OOH, (**c**) OH + OH, (**d**) OH + H + OH, and (**e**) 2H_2_O molecules binding to Cu_2_-N_8_/Gr (white, grey, blue, orange, and red spheres represent hydrogen, carbon, nitrogen, copper, and oxygen atoms, respectively).

According to the studies by Xiao et al. [34] and Noh et al. [45], the H atom could bind to the terminal O atom (Figure 7a) of the OOH intermediate to form an H_2_O molecule. In this study, the possibility of this reaction was determined via the optimization process. For all three temperatures, the final optimization resulted in the formation of a dual OH intermediate (Figure 7c and Figure 8c) instead of the formation of the water molecule. Figure 7 depicts the reaction at a temperature of 298.15 K. When the introduced H atom attempts to bond with the second O atom of OOH to form H_2_O, the previously bonded H atom breaks its bond with the second O atom and moves toward the first O atom (Figure 7b). During this process, a water molecule forms in the initial optimization steps, and then the Cu atom near the second O atom attracts it to establish a strong bond. This phenomenon is the same as that explained in previous H_2_O_2_ generation by the OOH intermediate. Due to electron depletion around the terminal O of *OOH, the O atom could not bear two H atoms as H_2_O. This could cause the initially bonded H atom to break its bond with the second O atom.

Figure 8 illustrates the optimized ORR steps, which differ from previous studies [28]. The changes observed in the 2OH and OH + H + HO intermediates, as opposed to O + H_2_O and OH +H_2_O in the ORR, may be attributed to the dual metal catalyst structure, as explained before. The following reaction Equations (1)–(5) depict the optimized ORR steps from the current study in an acidic medium. The asterisk (*) indicates the defect’s configuration.

* + O_2_ + 4H^+^ + 4e^−^→ *O_2_ + 4H^+^ + 4e^−^ (1)

*O_2_ + 4H^+^ + 4e^−^ → *OOH + 3H^+^ + 3e^−^ (2)

*OOH + 3H^+^ + 3e^−^→ *2OH + 2H^+^ + 2e^−^ (3)

*2OH + 2H^+^ + 2e^−^ → *(OH + H + HO) + H^+^ + e^−^ (4)

*(OH + H + HO) + H^+^ + e^− ^ → * + 2H_2_O (5)

### 2.3. Reaction Spontaneity

**Figure 9:**
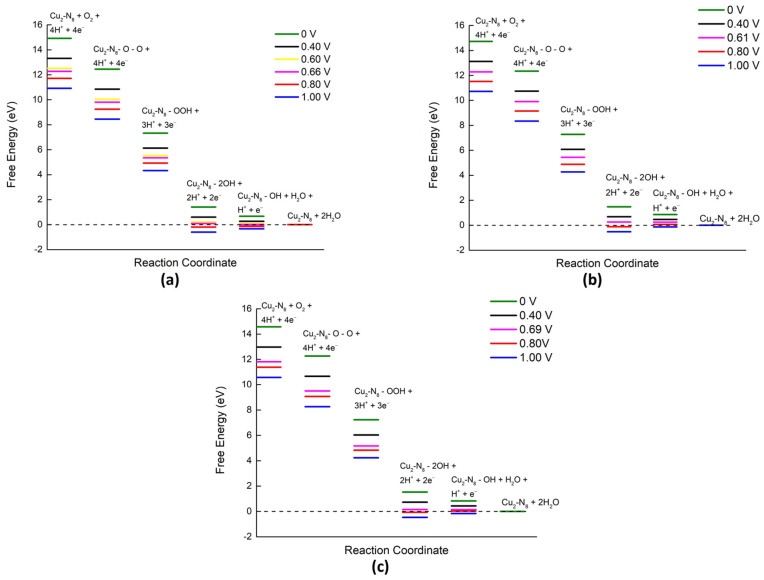
Free energy diagrams of Cu_2_-N_8_/Gr at different temperatures under various cell potentials in an acidic medium: (**a**) 298.15 K, (**b**) 353.15 K, and (**c**) 393.15 K (note: the OH + H_2_O intermediate is the same as the OH + H +OH intermediate, as explained previously).

All free energy calculations were performed for the ground state of the ORR steps, as shown in Figure 9. After conducting free energy calculations for all three temperatures, unlike in our previous studies [36], it was found that the maximum cell potential was greater than 0.60 V. At the initial temperature of 298.15 K, all reaction coordinates exhibited a downhill process (spontaneous process) until the cell potential reached 0.66 V (maximum cell potential). Beyond the maximum cell potential, the last step of the ORR became an uphill process (Figure 9a), indicating an increase in the energy barrier between the reaction coordinates. Further enhancement of cell potential is attributed to the initiation of the uphill process from the 2OH intermediate step. The difference in maximum cell potentials between the previous and current findings may be attributed to the inclusion of a dispersion correction for van der Waals forces in the Gaussian calculations of the current study.

By increasing the temperature to 353.15 K, the maximum cell potential decreased to 0.61 V (Figure 9b). Beyond 0.61 V, the chemical reactions become uphill processes from the 2OH intermediate step to the OH + H_2_O intermediate step. This adverse trend intensifies with higher temperatures. When the temperature was raised to 393.15 K, the maximum cell potential reached the highest value of 0.69 V (Figure 9c). For cell potentials higher than 0.69 V, the OH + H_2_O intermediate step acted as a barrier to the downhill process of the ORR. According to the free energy data, further increases in the cell potential augmented the uphill process between the 2OH and OH + H_2_O intermediates. The order of maximum cell potential at different temperatures was as follows: 353.15 K < 298.15 K < 393.15 K, indicating that temperature and maximum cell potential were not directly proportional. The decrease in maximum cell voltage at around 353.15 K suggests a decline in cell performance, which aligns with the findings of Ferraris et al. [19], who reported the lowest cell potential around the operating temperature of 343.15 K, supporting our observation. Furthermore, other investigations [15,16] suggest that at 393.15 K PEM fuel cell performance improved dramatically in congruence with cell voltage escalation due to high temperature.

In the free energy calculations, the observed up–down behavior of the maximum cell potential with temperature is mainly caused by OH + H_2_O intermediate step. The ESP diagram of the OH + H_2_O intermediate (Figure 2c) delineates that at 393.15 K, this intermediate gains high stability due to a dense and symmetric electron density distribution. This stability, along with higher electron mobility at higher temperatures, could contribute to a higher maximum cell potential observed at 395.15K compared to 353.15 K, a conclusion supported by HOMO–LUMO energy calculations [23,24].

The free energy analysis of the first and last steps in the ORR revealed a 15 eV gap, showing a departure from the typical values observed in conventional ORR catalysts. In a work by Kattel et al. [28], the free energy of O_2_ was determined through the reaction O_2_ + 2H_2_ → 2H_2_O, resulting in a free energy change of 4.92 eV. This value contrasts with most other density functional theory (DFT) calculations, where such values typically fall below 8 eV [27,46]. While a high free energy gap might suggest reduced effectiveness of the catalyst, insights from Santisouk et al. [47] challenge this notion. Binding energy, bond length, and HOMO–LUMO energy calculations indicate the effectiveness of the catalyst with intermediates. Notably, the introduction of dispersion correction (GD3BJ) in the current study contributes significantly to this observed change. In contrast, a previous study [36] of ours in the same research field did not exhibit such a substantial free energy gap, with the O_2_ to 2H_2_O free energy change hovering around 6 eV. This prompts consideration of alternative dispersion correction methods that could potentially enhance the accuracy of the free energy results.

Furthermore, given the novelty of the catalyst’s structure, additional studies are warranted to validate its effectiveness. It is worth noting that some ORR free energy calculations in the literature indicate differences between the first and last ORR steps ranging from 8 to 14 eV [34,47,48] emphasizing the variability in observed outcomes across different studies.

Table 4 presents the binding energy, formation energy, and bond length of molecules on the catalyst surface at the initial temperature for various catalyst types. All the catalysts under investigation are non-platinum group metal (non-PGM) types, featuring transition metals and nitrogen doping. In terms of formation energy, our current study yields the most promising results, with the cobalt (Co)-based catalyst displaying the least promising outcome. Specifically, the titanium (Ti)-based catalyst exhibits the strongest binding capability for O_2_ and H_2_O intermediates, suggesting its limited effectiveness as a catalyst material. Conversely, the Co-based catalyst demonstrates the weakest binding capability for the O_2_ intermediate, indicating its diminished efficacy as a catalyst. In the context of H_2_O_2_ formation at the conclusion of the ORR, our current study reveals no H_2_O_2_ formation, whereas all other catalysts exhibit such potential. Notably, the Co-based catalyst, characterized by its weakest binding strength and longest bond length, implies the likelihood of H_2_O_2_ formation and easy detachment from the catalyst layer. Furthermore, all dual molecules in our current study exhibit dual bonding, contributing to their strong attachment to the catalyst. These findings provide valuable insights into the diverse binding characteristics of the catalysts, shedding light on their potential efficacy in the context of the ORR.

Nonetheless, it is important to acknowledge several limitations of this study. Firstly, the use of a small 3–21G basis set may be considered a limitation. However, this choice was made to compare the results with previous investigations by Bhatt et al. [35], as well as to maintain consistency with our earlier study [36] and to compare with the Cu-N_2_/Gr results. Additionally, insufficient computational power meant that the study could not use a higher basis set. Furthermore, this study did not incorporate periodic boundary conditions (PBC), which distinguishes it from the approach employed by Xiao et al. [34]. The utilization of different DFT simulation software in these referenced studies precludes direct comparison with our findings. It should also be noted that the preference for Gaussian software over VASP or ab initio molecular dynamics simulation software was influenced by financial constraints and a lack of specialists. In this study, a single layer of graphene was employed to examine the impact of the defect structures on the surface of the catalyst in the context of ORR, a methodology similar to those of Bhatt et al. [35] and Kattel et al. [28]. In future studies related to this research, the use of the PBC approach will be explored to facilitate more direct comparisons with the current study and potentially expand the scope of investigation.

## 3. Methodology

The DFT calculations were performed using the Gaussian 09w software, applying the B3LYP method with a 3–21G basis set. The GD3BJ van der Waals correction (dispersion correction) was applied for every calculation. The form of the defect structures was visualized with GaussView 6.0. All atoms in each structure were relaxed using an optimization function in Gaussview 6 with the previously stated basis set and method. A single pristine graphene layer was created as the base, and from it, the Cu_2_-N_8_/Gr structure was developed to investigate the ORR under various temperatures (298.15, 353.15, and 393.15 K). The pristine graphene layer design at the initial temperature has C-C and C-H bond lengths of 1.43 and 1.08 Å, respectively, and a C-C-C angle of 119.92˚ which is compatible with the studies by Bhatt et al. and Hernandez et al. [35,49]. The structure that features the pyridinic nitrogen was constructed. Following the initial optimization, structures with a 1-unit imaginary frequency (=1) were reoptimized by using a stability function in GaussView 6. Individual optimization of the molecules involved in the ORR steps was carried out, with subsequent measurement and comparison of bond lengths and bond angles with previous studies by Xiao et al., Bhatt et al., and Balasooriya et al. [34,35,36] on this subject.

The stability of the catalyst structure with the cathode potential at different temperatures was confirmed. Stability was inspected using the formation energy (Δ*E*) defined as [28,35],

(6) $$\Delta E=E_{graphene+({Cu}_{2}-N_{8})}+y\mu_{C}-\left(E_{graphene}+x\mu_{N}+E_{M}\right)$$

Here, $E_{graphene+({Cu}_{2}-N_{8})}$ is the energy of the optimized Cu_2_-N_8_ graphene layer with the thermal correction to energy. $\mu_{C}$ and $\mu_{N}$ are the chemical potential of carbon, which is defined as the total energy per carbon atom for defect-free graphene, and the chemical potential of nitrogen, defined as half of the total energy of the N_2_ molecule with the thermal correction to energy, respectively. *x* represents the number of nitrogen atoms added, and *y* represents the carbon atoms removed during the defect formation. *M* is the metal atom (Cu) doped in the system. $E_{graphene}$ is the energy of the optimized pristine graphene layer with the thermal correction to energy. $E_{M}\;$ is the total energy of *M^n^*^+^ defined as

(7) $$E\left(M^{n+}\right)=E\left(M\right)-neU$$

where $E\left(M\right)$ is the total energy with the thermal correction to energy of isolated *M* (*M* = Cu) in the gas phase and *n*, *e*, and *U* are the number of electron transfer (+2), electron charge, and the external potential, respectively.

Binding energies (*BEs*) were calculated for the ORR steps in an acidic medium, as defined by [28,34,35],

(8) $$BE=E_{defect+molecule}-(E_{defect}+E_{molecule})$$

Here, $E_{defect+molecule}$ is the total energy with the thermal correction to the energy of molecules adsorbed by the defective graphene. $E_{defect}$ is the total energy with the thermal correction to energy of the defective graphene configuration, and $E_{molecule}$ is the energy of isolated molecule species (O_2_, H_2_O, 2H_2_O, OOH, OH, 2OH, H_2_O_2_) with the thermal correction to energy. Negative signed binding energies indicate energy released during the bonding process which is more favorable to the process. The formation of H_2_O_2_ during ORR is also considered.

The ORR steps of an ordinary catalyst in an H^+^ medium are defined as follows [28], where * indicates the defect’s configuration.

* + O_2_ + 4H^+^ + 4e^−^→ *O_2_ + 4H^+^ + 4e^−^ (9)

*O_2_ + 4H^+^ + 4e^−^ → *OOH + 3H^+^ + 3e^−^ (10)

*OOH + 3H^+^ + 3e^−^→ *O + H_2_O + 2H^+^ + 2e^−^ (11)

*O + H_2_O + 2H^+^ + 2e^−^ → *OH + H_2_O + H^+^ + e^−^ (12)

*OH + H_2_O + H^+^ + e^−^→ * + 2H_2_O (13)

The free energies were calculated for each ORR step of all defect configurations as defined by [28],

(14) $$\Delta G=\Delta E+\Delta ZPE-T\Delta S+\Delta G_{U}+\Delta G_{pH}+\Delta G_{field}$$

ΔE represents the energy calculated from DFT related to the relevant reaction step with the thermal correction to energies, while ΔZPE is the correction obtained from DFT calculations, accounting for the zero-point energy. The value of entropy (*S*) was obtained from the DFT calculations for the three temperatures (*T* = 298.15, 353.15, and 393.15 K) as a variable in this study, depending on temperature (*T*). In the context of this research, *ΔG_U_ = −eU* where *U* and *e* are the electrode potential and the charge transferred, respectively. Additionally, $\Delta G_{pH}=k_{B}T\times ln10\times pH$, where *k_B_* is the Boltzmann’s constant, and *T* is the temperature. Since this study considered acidic media, *pH =* 0. Therefore, normally the $\Delta G_{pH}$ value is considered to be zero. $\Delta G_{field}$ is the contribution of the interaction of adsorbate with the local electric field in the electric double layer formed in the vicinity of the cathode, which is negligible according to Nørskov et al. [50]. Corrections of zero-point energy and entropy values were obtained after frequency calculations. Free energy vs. reaction coordinate graphs were plotted with different potentials (*U*) for all temperature values.

The energy gap (*E*_g_), chemical hardness (*η*), chemical potential (*μ*), and electrophilicity index (*ω*) were calculated using Koopman’s principle for optimized structures as defined by [37,38,39,40,51,52],

(15) $$E_{g}=E_{LUMO}-E_{HOMO}$$

(16) $$\eta =\frac{I-A}{2}$$

(17) $$\mu =-\frac{\left(I+A\right)}{2}$$

(18) $$\omega =\frac{\mu^{2}}{2\eta }$$

Here, *I* and *A* are the ionization potential (≅−*E_HOMO_*) and the electron affinity (≅−*E_LUMO_*), respectively.

## 4. Conclusions

Leveraging the computational prowess of density functional theory, insights were gleaned into the structural dynamics of the Cu_2_-N_8_/Gr motif across three pivotal temperature gradients: 298.15, 353.15, and 393.15 K. Within these defined thermal windows, the motif exhibited an inherent predisposition toward spontaneous oxygen reduction reactions (ORRs), with a peak cell potential of 0.69 V recorded at an elevated threshold of 393.15 K. A discernible shift in the maximum cell potential was observed as temperatures progressed from 298.15 to 353.15 K, marking a transition from 0.66 to 0.61 V. Formation energy evaluations underscored the motif’s unwavering stability across a potential (*U*) spectrum ranging from 0 to 7.21 V, irrespective of the temperature in question. The maximum cell potential of the motif within the potential range of stability delineates better structural stability. Delving deeper into chemical metrics—spanning chemical hardness, potential, and electrophilicity—the motif showcased consistent ORR trajectories across the temperature continuum, with the singular exception of the OH + H_2_O (manifesting as OH + H +OH) intermediate. Intricate vibrational and electrostatic potential assessments pinpointed this intermediate as the pivotal agent that mediates cell voltage variations across disparate temperatures. Despite a subtle attenuation in ORR stepwise stability with surging temperatures, the overarching cell potential intriguingly reached its apogee at 393.15 K, indicating that catalyst performance had not been reduced. In fact, the HOMO–LUMO evaluations and IR spectrum reiterate this assertion, highlighting the motif’s unwavering stability across escalating temperatures. At the zenith of 393.15 K, the motif not only boasts unparalleled stability but also outperforms others in terms of cell potential, solidifying its credentials as a prime catalyst contender. The binding energy values slightly escalate with increasing temperature, except for the OH + H_2_O intermediate for the ORR steps. The binding dynamics of this intermediate witnessed a decline as temperatures transitioned from 298.15 K to 353.15 K, followed by an upswing at 393.15 K. Singular OH and H_2_O intermediates, due to the optimization dynamics, were found to be incongruent with conventional ORR pathways. Free energy assessments across the temperature spectrum showcased the motif’s spontaneous reactivity, consistently exceeding the 0.6 V cell potential threshold. Significantly, at 393.15 K, this potential reached an unparalleled 0.69 V, the pinnacle at all examined temperatures. To resolve the higher gap between the first and last ORR steps of the free energy data, further investigations should be carried out with different dispersion corrections and basis sets. Therefore, considering stability, binding strength, maximum cell potential, and spontaneity, the catalyst shows the best performance at 393.15 K, while its performance at 353.15 K appears sub-optimal, a sentiment echoed in the existing literature [15,16,19]. Similar to our previous work, this study also delineates a low probability of forming H_2_O_2_ at all temperatures in DFT optimization. Consequently, adherence to the quartet-electron pathways across all temperature spectra potentially preserves current density uniformity. In summary, the Cu_2_-N_8_/Gr catalyst, when assayed at 393.15 K, emerges as the gold standard, melding superior cell potential with minimized H_2_O_2_ formation.

## Figures and Tables

**Table 1:** Formation energies of the Cu_2_-N_8_/Gr structure determined at the temperatures (*T*) of 298.15, 353.15, and 393.15 K under various external potentials (*U*).

*T*/K	*U*/V	Formation Energy/eV
**298.15**	0	−28.856
	1	−24.856
	4	−12.856
	6	−4.856
	7.21	−0.016
	7.22	0.024
**353.15**	0	−28.865
	1	−24.865
	4	−12.865
	6	−4.865
	7.21	−0.025
	7.22	0.015
**393.15**	0	−28.864
	1	−24.864
	4	−12.864
	6	−4.864
	7.21	−0.024
	7.22	0.016

**Table 2:** Energy gap (*E*_g_), chemical hardness (*η*), chemical potential (*μ*), and electrophilicity index (*ω*) values of the O_2_, OH + H_2_O, 2H_2_O intermediates, and defect structures for different temperatures (*T*).

*T*/K	Intermediate/Structure	*E*_g_/eV	*η*/eV	*μ*/eV	*ω*/eV
298.15	Cu_2_-N_8_/Gr	0.992	0.496	−3.469	6.063
	O_2_	0.738	0.369	−3.615	8.854
	OH + H_2_O	0.947	0.473	−3.358	5.953
	2H_2_O	0.686	0.343	−3.322	8.048
353.15	Cu_2_-N_8_/Gr	0.992	0.496	−3.469	6.063
	O_2_	0.738	0.369	−3.615	8.854
	OH + H_2_O	0.777	0.388	−3.418	7.520
	2H_2_O	0.686	0.343	−3.322	8.048
393.15	Cu_2_-N_8_/Gr	0.992	0.496	−3.469	6.063
	O_2_	0.738	0.369	−3.615	8.854
	OH + H_2_O	1.039	0.519	−3.412	5.605
	2H_2_O	0.686	0.343	−3.322	8.048

**Table 3:** The binding energies (BEs) of the Cu_2_-N_8_/Gr structure at different temperatures and the shortest distance (*d*) between Cu-O and O-O atoms in angstroms (Å).

Temperature/K	Molecule	BE/eV	*d*_O-O_/Å	*d*_Cu-O_/Å
**298.15**	O_2_	−3.00	1.471	1.893
	OOH	−9.60	1.565	1.887
	OH + OH	−10.97	2.680	1.864, 1.809
	OH + H_2_O	−5.35	2.441	1.960, 1.939
	OH	−6.45	-	1.832
	H_2_O	−1.11	-	2.144
	2H_2_O	−2.12	2.519	1.997, 2.909
**353.15**	O_2_	−2.99	1.471	1.893
	OOH	−9.59	1.565	1.887
	OH + OH	−8.51	2.680	1.864, 1.809
	OH + H_2_O	−5.25	-	1.943, 1.946
	OH	−0.25	-	1.837
	H_2_O	−1.09	-	2.144
	2H_2_O	−2.11	2.519	1.997, 2.909
**393.15**	O_2_	−2.98	1.471	1.893
	OOH	−9.58	1.565	1.887
	OH + OH	−8.50	2.680	1.864, 1.809
	OH + H_2_O	−5.33	2.437	1.946
	OH	−0.24	-	1.837
	H_2_O	−1.08	-	2.144
	2H_2_O	−2.10	2.519	1.996, 2.908

**Table 4:** Comparison of binding energy (BE), bond length of molecule to catalyst (*d*_M-O_), and formation energy at zero potential (*E*_F(U = 0)_) of different catalysts in this study with the published studies, at initial temperature. M-O is defined as M = metal atom in catalyst surface and O = oxygen atom of molecule.

Catalyst	Molecule	BE/eV	*d*_M-O_/Å	*E*_F(U = 0)_/eV	References
**Cu-N_2_/Gr**	O_2_	−2.90	2.047	−5.68	
	OOH	−1.81	1.957		
	O	−5.14	1.890		Bhatt et al. [35]
	OH	−2.68	1.908		
	H_2_O	−1.08	2.203		
	H_2_O_2_	−1.49	2.236		
**Cu-N_4_/Gr**	O_2_	−2.76	1.98	−13.22	
	OOH	−1.70	1.87		
	O	−5.15	1.88		Balasooriya et al. [36]
	OH	−1.99	1.84		
	H_2_O	−1.23	2.08		
	H_2_O_2_	−0.92	2.18		
**Co-N_4_/Gr**	O_2_	−0.67	1.93	−3.56	
	OOH	−1.02	1.87		
	O	−3.18	1.71		Kattel et al. [28]
	OH	−2.44	1.88		
	H_2_O_2_	−0.06	3.36		
**Cu_2_-N_8_/Gr**	O_2_	−3.00	1.893	−28.856	Current study
	OOH	−9.60	1.887		
	OH + OH	−10.97	1.864, 1.809		
	OH + H_2_O	−5.35	1.960, 1.939		
	OH	−6.45	1.832		
	H_2_O	−1.11	2.144		
	2H_2_O	−2.12	1.997, 2.909		
	H_2_O_2_	None forming	None forming		
**Ti-N_4_/Gr**	O_2_	−8.95	1.838	−3.72	
	OOH	−7.99	1.829		
	O	−12.06	1.644		Bhatt et al. [35]
	OH	−7.99	1.771		
	H_2_O	−4.14	2.067		
	H_2_O_2_	−9.13	1.829		

## Data Availability

All data used to support the findings of this study are included in the article.
